# Elimination of schistosomiasis: the tools required

**DOI:** 10.1186/s40249-017-0370-7

**Published:** 2017-11-20

**Authors:** Robert Bergquist, Xiao-Nong Zhou, David Rollinson, Jutta Reinhard-Rupp, Katharina Klohe

**Affiliations:** 1 Ingerod, Brastad, Sweden; 2 National Institute of Parasitic Diseases Chinese Center for Disease Control and Prevention, Shanghai, 200025 China; 3 Department of Life Sciences, The Natural History Museum, London, SW7 5BD UK; 4 Merck, Coinsins, Switzerland; 5 Global Schistosomiasis Alliance, Westenriederstrasse 10, 80331 Munich, Germany

**Keywords:** Schistosomiasis, Tools, Morbidity control, Elimination, Transmission, Strategy, Surveillance, Global alliance

## Abstract

**Background:**

Historically, the target in the schistosomiasis control has shifted from infection to morbidity, then back to infection, but now as a public health problem, before moving on to transmission control. Currently, all endemic countries are encouraged to increase control efforts and move towards elimination as required by the World Health Organization (WHO) roadmap for the global control of the neglected tropical diseases (NTDs) and the WHA65.21 resolution issued by the World Health Assembly. However, schistosomiasis prevalence is still alarmingly high and the global number of disability-adjusted life years (DALYs) due to this infection has in fact increased due to inclusion of some ‘subtle’ clinical symptoms not previously counted.

**Main body:**

There is a need to restart and improve efforts to reach the elimination goal. To that end, the first conference of the Global Schistosomiasis Alliance (GSA) Research Working Group was held in mid-June 2016 in Shanghai, People’s Republic of China. It reviewed current progress in schistosomiasis control and elimination, identified pressing operational research gaps that need to be addressed and discussed new tools and strategies required to make elimination a reality. The articles emanating from the lectures and discussions during this meeting, together with some additional invited papers, have been collected as a special issue of the ‘*Infectious Diseases of Poverty*’ entitled ‘Schistosomiasis Research: Providing the Tools Needed for Elimination’, consisting of 26 papers in all. This paper refers to these papers and discusses critical questions arising at the conference related to elimination of schistosomiasis.

**Conclusion:**

The currently most burning questions are the following: Can schistosomiasis be eliminated? Does it require better, more highly sensitive diagnostics? What is the role of preventive chemotherapy at the elimination stage? Is praziquantel sufficient or do we need new drugs? Contemplating these questions, it is felt that the heterogeneity of the endemic areas in the world requires WHO policies to be upgraded instituting new, differentiated guidelines.

**Electronic supplementary material:**

The online version of this article (doi: 10.1186/s40249-017-0370-7) contains supplementary material, which is available to authorized users.

## Multilingual abstracts

Please see Additional file 1 for translations of the abstract into the five official working languages of the United Nations.

## Background

Large-scale, repeated treatment with praziquantel has resulted in a lasting improvement with respect to the pathology associated with schistosomiasis, and in many areas the control strategy is shifting from targeting morbidity to elimination of the infection as a public health problem. Nonetheless, the worldwide schistosomiasis prevalence remains as high as ever and the estimated number of disability-adjusted life years (DALYs), an important measure used to assess burdens of disease [1], has actually increased. This latter effect is, however, due to inclusion of some previously under-recognized morbidities, e.g., growth stunting, anaemia and retarded intellectual development not accounted for in the DALY score before. This has imposed a reconsideration of the impact of this disease and schistosomiasis now comes second on the list of the now 18 neglected tropical diseases (NTDs) issued by the World Health Organization (WHO) [2, 3] and would have been first, had the intestinal nematodes not been presented together.

For the first time, there is now a real belief in the possibility of schistosomiasis elimination as expressed in Resolution 65.21 of the World Health Assembly (WHA65.21). WHO’s latest schistosomiasis fact sheets emphasize the need to reach the goal of regular treatment with praziquantel of at least 75% of children by 2020 [4]. In addition to provision of essential medicines, water, sanitation and hygiene (WASH) recommended in the WHO roadmap for the global control of the NTDs [5], new complimentary drugs, local recommendations for snail control, surveillance/management of hotspots and, above all, a switch to more sensitive diagnostics are now urgently needed. Although stool examination (for intestinal schistosomiasis) and urine filtration (for the urogenital form of the disease) remain adequate for areas where the burden of disease is high, disease intensities in most parts of the endemic world have receded thanks to repeated chemotherapy and are now approaching the limits of sensitivity of these techniques in some endemic areas. This results in underestimation of prevalence and contributes to difficulties in the assessment of the impact of praziquantel. Daily variations in host egg excretion, particularly pronounced in *Schistosoma japonicum* infections [6], is an added drawback increasing the number of false negative tests in low-intensity infection areas. Thus, approaches that worked well in the past are less suitable when the target shifts from control of morbidity to interruption of transmission. Indeed, this pivotal juncture has been reached in some countries, where elimination of schistosomiasis within the next decade is now a distinct possibility [7]. In this perspective, various questions need to be brought forward. For example, while it would be useful to integrate schistosomiasis control with that of the soil-transmitted helminths in hypo-endemicity areas, the introduction of a surveillance-and-response system [8] supported by sensitive diagnostic tools is required in all areas where transmission control is being launched.

## All together now

The first conference of the Research Working Group of the Global Schistosomiasis Alliance (GSA) (http://www.eliminateschisto.org), held 14–15 June 2016 in Shanghai, People’s Republic of China (PR China), reviewed current progress in schistosomiasis control. Pressing operational research gaps were identified while discussing the new tools and strategies required to make elimination a reality [9]. For example, it was felt that a highly sensitive diagnostic tool is not only necessary for appropriately targeting populations requiring chemotherapy, but would also facilitate the recognition of the full distribution and extent of damage inflicted by this disease. Lectures and discussions taking place at the conference together with some additional invited papers make up the bulk of this special issue that contains 26 papers in all. A few general-purpose papers come from the United States, PR China and Europe but most papers emanate from the African continent reflecting the fact that this part of the world has the largest endemic areas and harbours more than 90% of all known schistosomiasis cases [10]. The articles discuss special issues with relevance to schistosomiasis in various African and Southeast Asian countries. Missing correspondence from Brazil, Egypt and The Philippines would have completed the published overview. Although most contributions deal with the three major species in general, five papers specifically focus on *S. haematobium*, four on *S. japonicum* and one paper each on *S. mansoni* and *S. mekongi*.

**Fig. 1:**
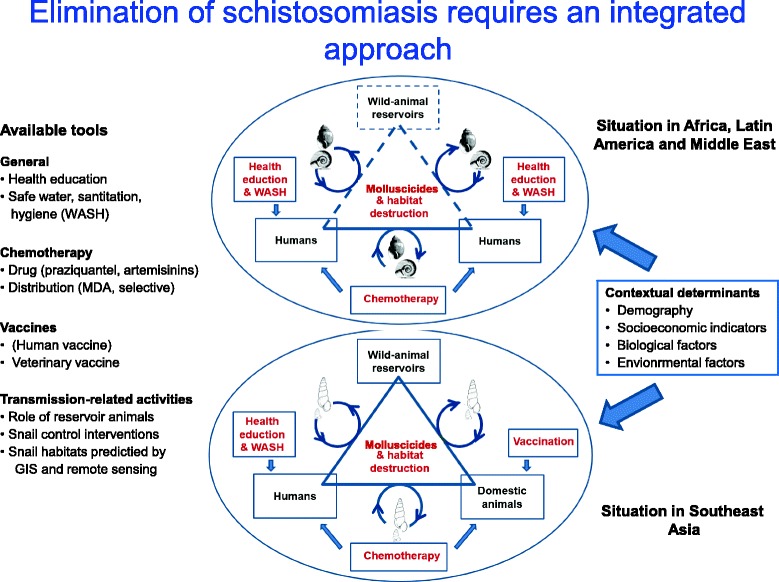
Schematic figure depicting important differences between the major endemic areas and the applicable control tools. In the Africa, Latin America and Middle East, the parasites (*S. mansoni* and *S. haematobium*) depend on pulmonate snails (*Biomphalaria* and *Bulinus)* for transmission, which is confined to human/human transfer in principle, while Asian schistosomiasis (due to *S. japonicum* or *S. mekongi* and the amphibious *Oncomelania* and freshwater *Neotricula snails*) is zoonotic with many animal reservoirs

Areas endemic for schistosomiasis are highly heterogenic, partly due to the different schistosome species involved and partly due to the various geographical environments encountered. This makes it difficult to divide the world into specific areas where specific control techniques can be applied. For example, while the sub-Saharan scene is not unlike that of The Philippines (presumably because both areas have perennial transmission and the socioeconomic level in the rural areas is similar), control activities vary due to the different schistosome species involved. On the other hand, while snail control is easier to achieve in PR China and The Philippines compared to Africa and Brazil (due the fact that *Oncomelania,* the snail intermediate host in the former countries is amphibious, while the pulmonate snail species playing this role elsewhere are not), the tendency of *S. japonicum* to infect a large number of animal hosts besides humans, is an added obstacle in Southeast Asia (see Fig. 1).

### Farewell to the ‘God of Plague’

Ever since Mao Zedong’s poetic battle cry of 30 June 1958 against schistosomiasis [11], PR China has relentlessly worked on limiting the impact of schistosomiasis. The core areas that still remain endemic for *S. japonicum* after 60 years’ control activities consist now mainly of the marshlands around the Dongting and Poyang lakes in central China and along the Yangtze River downstream from Dongting Lake [12]. Three papers by Sun and colleagues [13–15] provide new insights into the development of improved strategies for transmission interruption in these areas. They review work done from the time of the millennium shift up to the present day and discuss a roadmap for elimination based on integrated environmental improvement of the marshlands carried out through the implementation of industrial, agricultural and resource developments along the Yangtze River. The industrial projects included the building of boat factories, docks and harbors; the agricultural projects consisted of land reclamation; and the water resource projects produced hard river banks lined with concrete and new sluices, including snail-retention pools. This approach, representing long-term intersectoral interventions involving governmental departments of health, of agriculture, of water resources and of land forestry, has not only reduced the snail habitats but also contributed to an acceleration of the socio-economic development in the area now shown to be capable of a sustaining continued reduction of transmission.

Although of less severity then the Yangtze River Basin with respect to schistosomiasis endemicity, the mountainous areas in eastern China are a major problem since the topography impedes large-scale approaches requiring somewhat different strategies than those used elsewhere. Liu et al. [16] report good results from a 10-year longitudinal study evaluating a small-scale approach where an important ingredient is the replacement of bovines with machines. Chemotherapy, snail control and sanitation were also used and information, education and communication (IEC) materials were distributed to each household.

Other papers took a more methodological view on the epidemiology of schistosomiasis in PR China, e.g., Hu et al. [17] who assessed environmental factors associated with transmission, and Xia et al. [18] who emphasized the importance of spatial distribution and temporal correlation when designing an effective surveillance strategy. The former research group, focussing on environmental variables found proximity to Yangtze River having the strongest effect on sustaining schistosomiasis followed by the number of daylight hours. This is hardly surprising but the main conclusion that interactions between factors with weak influence on their own can play an important role when combined, is novel. The importance of environmental factors was followed up by the latter group emphasizing the spatial distribution and temporal correlation of infected cases when designing an effective surveillance strategy. They sampled faeces from domestic animals in the fields around a number of villages along the Yangtze River and determined the proportions of *Schistosoma*-infection in these samples to establish a prediction matrix for each village that could be used to sort them into different types of cluster. These findings may have important implications for control of schistosomiasis since the spatial aggregation found can be used as basis for targeted measures.

### The heart of the matter

Even if serious pathology due to schistosomiasis has largely been overcome thanks to preventive chemotherapy, sub-Saharan Africa still includes large areas with high-intensity infection dynamics. Risk factors associated with urinary schistosomiasis in school children, investigated in Cameroon [19] and in Zambia [20] demonstrate that local transmission is strongly associated with gender and age with the highest intensity of infection in 10–15 years olds corroborating what is reported in other parts of Africa, e.g., by Tingley et al. [21]. Interestingly, Stensgaard et al. [22] found intensity of infection to be slightly more pronounced at higher altitudes, which goes against the belief that low night-time temperatures inhibit transmission by affecting snail reproduction negatively. However, although not stated exactly in meters, it seems that the altitudes in Zambia mentioned in the paper by Simoonga et al. [20] were not as extreme as those previously investigated in Uganda [23] and now again in the paper contributed for this special issue by Stanton et al. [24]. Both the latter research groups assessed altitudes up to several thousand meters in Uganda. Regardless of the strength of transmission at these levels of altitude, spatial epidemiological predictions predict that some extra six million people must be added to the at-risk population in Uganda, a fact that should be acted upon in all communities located at high plateaus (in Uganda as well as in other countries) that may currently fall outside national control activities.

A well-known transmission focus in Cameroon, with both urogenital schistosomiasis and soil-transmitted helminthiasis (STH), recently subjected to several control interventions was reinvestigated by Campbell et al. [25]. With respect to STH they found only low levels of infection in those tested, while there was high-intensity schistosomiasis in many places. Despite an improved WASH-related infrastructure, water contact risk scores were higher in some places and in one area significantly more women reported signs and symptoms associated with female genital schistosomiasis. This assessment highlights the importance of understanding that the epidemiological dynamics that support STH are less powerful when it comes to schistosomiasis, which can often appear intransigent. Further studies are needed to evaluate intensified interventions in terms of gaining and sustaining control of transmission of both these two groups of helminth infection, which often have overlapping endemic areas.

## Can schistosomiasis be eliminated?

Long-term, well-structured control programmes make a difference. The Japanese control programme is proof that not only elimination, but even eradication in country, can be achieved by long-term, uninterrupted activities. As made clear by Kajihara and Hirayama [26], once the epidemiology had been determined, elimination was a straightforward affair with the last new human infection reported in 1977 [27]. Encouraged by this achievement and the more recent progress in many other countries, PR China, Brazil, the Caribbean, Egypt and Morocco in particular, WHO recommends endemic countries to intensify control interventions, strengthen surveillance and initiate elimination campaigns with a view ultimately to break the transmission of this disease [28].

**Fig. 2:**
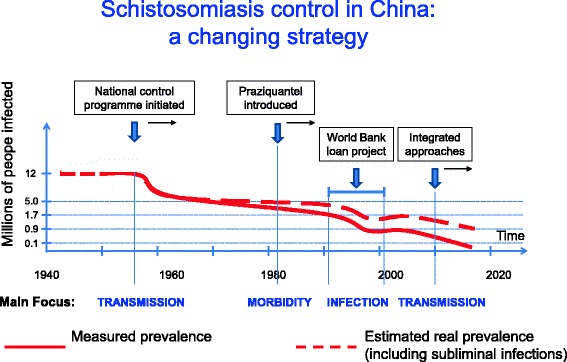
Overview of the Chinese control approach, originally based on snail control and a ‘find and treat’ strategy but redirected to MDA with praziquantel from the 1980s. The current strategy is based on integrated control strategy, including snail control, chemotherapy and WASH, but it also includes the promotion of tractors for fieldwork and deployment of transmission-blocking vaccination of water buffaloes and cattle in pilot areas

By reducing the number of infected humans in the country from around 12 million to less than 100,000 after more than 60 years of dedicated activities, the national schistosomiasis control programme in China may the best example of successful planning and execution of interventions targeting schistosomiasis (Fig. 2). However, also countries with less serious epidemiological problems initially, e.g. Morocco, have mounted control programmes and are now close to eradication of the disease. Sub-Saharan Africa [10] and The Philippines [29] currently represent the biggest challenges.

Today’s cornerstone for worldwide control of schistosomiasis consists of preventive chemotherapy using praziquantel delivery through mass drug administration (MDA) in areas known to be at risk for infection and disease. However, with at least 218 million people requiring preventive treatment in 2015 and 66.5 million people treated, this disease must be deemed to have a low drug coverage compared to many other NTDs [4]. This notwithstanding, Morocco and Oman are already at the surveillance-and-response stage as discussed in five articles in this special issue dealing with the possibility of actually eliminating the disease there within the next few years [30, 31]. However, the achievements made have to be sustained and the transition from infection control to transmission control requires guidelines how to proceed [32]. Suitable metrics derived from observed prevalence rates and compared with predetermined thresholds provide insights into exposure-related dynamics [33]. It will also be important to certify areas where transmission has been interrupted and how to institute a reasonable ‘survey-and-response strategy’ [8]. To be successful in the long term, elimination strategies must be sustained and tailored to all levels including the local situation. However, guidelines will presumably have to be geographically adjusted as the various schistosome species respond in somewhat different ways to preventive chemotherapy.

Transmission of schistosomiasis (*S. haematobium*) in Morocco has existed since historic times but is reported to have been prevented in 2004 and onwards [34]. To hinder re-emergence, subjects with positive antibody serology are required to be tested for active infection with sensitive tests. Indeed, two people cured 21 and 32 years ago, who remained with positive antibody serology, were finally shown to still have low levels of the highly specific *Schistosoma* circulating anodic antigen (CAA) [31], a fact reported at the conference as an indication that surveillance must go on for a long time, even indefinitely, in previously endemic areas. In contrast to Morocco, schistosomiasis (*S. mansoni*) in Oman was first reported in 1979 [35], although it might well have existed earlier. Recent data indicate a progressive prevalence decline throughout the 1980s and the 1990s and the disease is now said to have been eliminated [30]. However, further investigations with sensitive diagnostics along the lines used in Morocco [31] would be required to certify this.

Certain areas stand out as possible targets for elimination due to restricted geographical distribution of the disease. Indonesia represents a case in point where the endemic areas are confined to three small, isolated valleys in Central Sulawesi. As of 2006, integrated control had reduced the human prevalence to 0.5% - 1% but more recent data indicate that these levels may not be holding [36]. Another example is that of *S. mekongi* in a Lao People’s Democratic Republic (Lao PDR) and Cambodia, where the disease is similarly constrained and elimination therefore seemingly feasible [28, 37]. The latest diagnostic intervention using high-definition diagnostics showed considerably higher levels of infection than estimated previously confirming a large underestimation of the prevalence of active schistosomiasis mekongi [38]. Hence, sustained control efforts are still needed to break transmission of *S. mekongi*.

### The case for high-definition diagnostics

Delineation of prevalence and intensity of infection based on non-equivocal documentation is a necessary step for elimination of schistosomiasis and success depends crucially on availability of highly sensitive diagnostic techniques. Historically, schistosomiasis diagnosis has relied on urine filtration for urogenital infection [39] and the modified Kato technique [40] for the intestinal type of the disease. Efforts to replace these microscopy methods with something better have not borne fruit until recently and it is due to two developments: 1) large-scale implementation of MDA with low-intensity infections as a consequence; and 2) availability of reliable high-definition techniques measuring the schistosome circulating cathodic antigens (CCA) and anodic antigens (CAA), both in serum and urine. There is much to be gained by switching from microscopy to testing for circulating antigens [41]. For example, as argued by Colley et al. [42] national control programmes faced with what appears to be an increasing population of egg-negative but worm-positive schistosomiasis cases find it difficult to decide which guidelines and strategies to enact. Indeed, the multiplicative portion of the life-cycle of schistosomes, in the snail intermediate host, favours continued transmission as long as even a few people maintain low numbers of worms passing eggs in their excreta [42]. A practical method for this kind of diagnosis is the recently developed high-sensitivity genus-specific *Schistosoma* CAA strip test, which has been considered for applicability in a strategy where large number of urine samples can be tested collectively after pooling, thus providing an ‘areal diagnosis’ [43]. Testing for CCA and CAA, both in urine and serum, in Lao PDR and Cambodia in conjunction with stool examination showed that detection of circulating schistosome antigens is on average about eight times more sensitive than egg detection [39].

With the increase of trade and communication between PR China and Africa, Chinese visitors have repeatedly been returning from Africa with schistosomiasis. It has been proposed to develop new non-species-specific screening tools and/or modify existing immunoassays. Two papers discuss the application of highly sensitive rapid assays for animal diagnosis, the colloidal gold immunochromatography [44] and the nested polymerase chain reaction (PCR) assay [45]. Veterinary tests are needed in China since bovines and goats are believed to be the main transmission sources there. Despite some degree of cross-reactions with other parasites, such as *Haemonchus* sp., *Orientobilharzia* sp., both assays proved useful for testing both wild and domestic animals, which will be increasingly needed after the infection has been eliminated from the human population.

### Current prospects for chemotherapy

Importantly, long-term repeated drug treatment induces selective pressures on parasites, which may lead to development of resistance. To find out how this risk could be minimised, Kabuyaya et al. [46] investigated responses to drug treatment in a limited sample of humans. They assessed the efficacy of praziquantel, determined re-infection and incidence rates of *S. haematobium* infection among school-going children where the prevalence at baseline amounted to 38%, almost entirely consisting of high-intensity infections. The average cure rate of 88% was recorded 4 weeks after the initial treatment, while the egg reduction rates were low suggesting a reduced praziquantel efficacy. Some degree of immunity may have developed and reflected by the overall reinfection rate of 8% that was the same both at 20 and 28 weeks post treatment.

Praziquantel treatment has been found to decrease adult-worm burden resulting in an even larger negative impact on the daily number of miracidia [47]. Such reductions in worm fecundity, evident already after low doses of praziquantel, suggest that egg-based diagnostics could overestimate the short-term drug effect with important implications for transmission control including the potential for undetected selection of resistance.

Pre-school children used to be excluded from the recommended target population for MDA and there was at first no encouragement to produce a formulation adjusted for treatment of children. When WHO changed recommendations to include pre-school children in 2010, the drawbacks of using crushed or divided tablets became apparent. Noting the knowledge gaps pertinent to the successful control of schistosome infection and disease in pre-school children, the target product profile for paediatric praziquantel as discussed by Mduluza and Mutapi [48], Reinhard-Rupp [49] has provided an update on the development of an innovative child-friendly orodispersible formulation proposed by the Pediatric Praziquantel Consortium (http://www.pediatricpraziquantelconsortium.org). The orodispersible formulation is currently based on the racemate- and enantiomer-pure praziquantel which are both being tested in comparison during an ongoing clinical Phase 2 study in endemic areas. After completing the full clinical development, the Pediatric Praziquantel Consortium aims to submit the regulatory dossier for WHO prequalification and for subsequent marketing approval in endemic countries with an expected product launch of the preparation for schistosomiasis paediatric case management in key endemic countries by 2020.

There is an urgent need for new drugs against this disease whose control entirely depends on this single drug that has been widely used for 40 years. Drugs developed and already approved for other diseases have sometimes been found to include antischistosomal properties and are therefore candidates for use also for other diseases than what they were originally intended for. Such potentially ‘repositioned’ drugs do not only include artemisinins used against malaria, but also a wide variety of other drugs. Importantly, they provide a shortcut to clinical trials as they would rapidly pass the regulatory authorities. Another avenue to follow is the continued search for new antischistosomal properties in plants. Bergquist et al. [50] summarize recent progress made in this field arguing that supplementing praziquantel with new antischistosomals targeting different parasite development stages would not only increase efficacy but also reduce the risk for drug resistance.

### Where is the vaccine?

As it does not prevent re-infection, praziquantel will not be particularly useful when we approach the elimination goal, while the work on schistosomiasis vaccines remains in a continuing uphill battle. Delayed vaccine development is mainly due to limited funding resulting from the general conviction that elimination can be reached without it, but reflects also the formidable immunological challenges opposing its realization. Not only has it been difficult to produce a vaccine mounting a strong, specific response against schistosomes, but safety aspects require a simultaneous restraint that is difficult to meet: reduction of the host’s immune responses against eggs trapped in the tissues from previous infections. Nevertheless, a few human schistosomiasis vaccine candidates have reached the stage of clinical trials [51], while a transmission blocking vaccine has already produced practical results in buffaloes and cattle in PR China [52]. It is therefore likely that the deployment of a transmission-blocking vaccine can soon be used as part of an integrated approach for the prevention, control and eventual elimination of schistosomiasis in areas where the infection is zoonotic, i.e. in Southeast Asia. Certification of a human schistosomiasis vaccine will take longer and the costs of development may be prohibitive.

## Conclusions

While PR China is moving towards elimination of schistosomiasis in the immediate future, the situation in sub-Saharan Africa is fundamentally different due to the shifting dynamics and heterogeneity of the disease. Jordan’s review of early efforts in controlling schistosomiasis in Africa [53] highlights discoveries and ideas of little practical relevance then, but of significance now when new tools are available. He notes that there has been little change in the overall concept of control, while a better understanding of the epidemiology has clarified what the objective of control should be. Taken up by today’s generation of researchers, his thoughts influence the current interest in the role of the environment and its effect on infection intensity establishing sustained and community-based surveillance schemes.

The central outcome of this two-day, international meeting was an agreement on the need to better tailor preventive chemotherapy to the local environment in endemic areas and to emphasize the use of other measures in addition to chemotherapy. It was felt that it would be useful to learn more about transmission dynamics by increased data collection, mapping, and intervention trials for high-transmission hotspots as well as encouraging surveillance and response approaches when low levels of infection intensity become generalized. Microscopy diagnosis should be switched to high-sensitivity approaches in areas characterized by low levels of infection without delay and WHO policies should be upgraded and geared at meeting the challenges of elimination of schistosomiasis with differentiated guidelines for morbidity control and elimination.

Recent progress in schistosomiasis control has generally been strong but also geographically uneven - many countries have been implementing preventive chemotherapy for several years with others still striving to achieve national coverage. While large areas are at the threshold of elimination, others show intransient hotspots. The gap between drug coverage needed and the current distribution of praziquantel remains inacceptable, particularly with respect to pre-school children and in Africa.

Accreditation protocols defining interruption of transmission are needed now and they should rest on a combination of antigen and antibody detection coupled with snail diagnostics in diverse testing ranges based on spatial statistics. High-definition diagnostic assays have the sensitivity and standardized application needed for the realignment of the three WHO levels of prevalence used to determine which control approach to put into operation. Basing the cut-off values for these levels on the levels of circulating schistosome antigens would represent no small advance and it could be set up already today.

## Additional file

Additional file 1: Multilingual abstracts in the five official working languages of the United Nations. (PDF 666 kb)

## Abbreviations

CAA: Circulating anodic antigen
CCA: Circulating cathodic antigen
DALYs: Disability-adjusted life years
GSA: Global Schistosomiasis Alliance
IEC: Information, education and communication
Lao PDR: Lao People’s Democratic Republic
PR China: People's Republic of China
MDA: Mass drug administration
NTDs: Neglected tropical diseases
PCR: Polymerase chain reaction
STH: Soil-transmitted helminthiasis
WASH: Water, sanitation and hygiene
WHO: World Health Organization
