# Supplementary material for: Elimination of schistosomiasis: the tools required
Source: Infect Dis Poverty. 2017 Nov 20;6:158. doi: 10.1186/s40249-017-0370-7 (PMC5694902; doi:10.1186/s40249-017-0370-7)
Supplement: Additional file 1: — Multilingual abstracts in the five official working languages of the United Nations. (PDF 666 kb) [file 40249_2017_370_MOESM1_ESM.pdf]

Translation of the abstract into the five official working languages of the United Nations

الأدوات المطلوبة للقضاء على مرض البلهارسيا

Robert Bergquist, Xiao-Nong Zhou, David Rollinson, Jutta Reinhard-Rupp, Katharina Klohe

الملخص
بالرغم من الجهود العالمية المبذولة للحد من انتشار مرض البلهارسيا ألا ان نسبة الإصابة بالمرض لا تزال مرتفعة على نحو مقلق وعالميا زاد عدد سنوات العمر المعاقة بسبب هذا المرض وهذا علي الرغم من إدراج بعض الأعراض السريرية الطفيفة التي يتم إحتسابها الان عند حساب معدل سنوات العمر المعاقة بسبب المرض ونظرا لإختفاء معدلات انتشار المرض الحادة في العديد من البلدان فقد تغيرت الاستراتيجية الحالية من استهداف نسبة المرض من أجل تقليلها إلي القضاء علي المرض نهائيا كمشكلة صحية عامة وذلك للوفاء بخارطة الطريق التي وضعتها منظمة الصحة العالمية (WHO) للمكافحة العالمية للأمراض الاستوائية المهملة (NTDs) وطبقا لقرار WHA65.2 وفي ظل هذه التطورات تم عقد المؤتمر الأول لتحالف مجموعة الفريق البحثي العالمي للبلهارسيا بهدف البحث والتطوير فيما يتعلق بالمرض والذي تم عقده في يونيه عام 2016 في مدينة شانغهاي بجمهورية الصين الشعبية وتم إستعراض التقدم المحرز حاليا في مكافحة البلهارسيا وتحديد أهم الثغرات البحثية في البحوث التطبيقية ومناقشة الأدوات الجديدة والاستراتيجيات المطلوبة للقضاء علي المرض علي أرض الواقع وتم جمع المواد المنبثقة من المحاضرات والمناقشات خلال هذا المؤتمر جنبا إلى جنب مع بعض الأوراق البحثية لعدد من الباحثين المدعوين وتم وضعها في عدد خاص تحت عنوان "بحوث البلهارسيا: توفير الأدوات اللازمة للقضاء على المرض" ويحتوي هذا العدد علي 27 ورقة بحثية وتمت فيه معالجة العديد من الأسئلة الحرجة ذات الصلة بالقضاء على المرض في الاجتماعات التي أجريت مثل هل يمكن القضاء علي البلهارسيا؟ هل نحتاج إلي عملية تشخيص عالية الحساسية؟؛ ما هو دور العلاج الكيميائي الوقائي في مرحلة القضاء علي المرض؟؛ هل نحن بحاجة إلى أدوية جديدة بالإضافة إلى البرازكوانتيل؟ والعديد من الأسئلة الأخرى وكانت المحصلة هي التوصية بحاجة منظمة الصحة العالمية لوضع مبادئ وسياسات توجيهية جديدة نظراً لعدم تجانس المناطق الموبوءة في العالم.

Translated from English version into Arabic by Mohamed R. Habib

消除血吸虫病需要的工具

Robert Bergquist, Xiao-Nong Zhou, David Rollinson, Jutta Reinhard-Rupp, Katharina Klohe

摘要
尽管全球都在努力遏制血吸虫病，但是其患病率仍然非常高，而且全球的血吸虫病伤残调整寿命年呈现上升。当然，伤残调整寿命年的增加是由于将一些轻微的临床症状也纳入了评价，但在很多国家重症病人已经消失了。因此，当前的策略已经从控制发辫转向消除（作为一个公共卫生问题）以对应 WHO 的全球控制被忽视热带病路径图和 WHA65.21 号决议。这些进展促使全球血吸虫病联盟研究工作组于 2016 年 6 月在中国上海第一次召开关于血吸虫病研究和发展的会议。会议审阅了血吸虫病控制的最新进展，发现了应用型研究方面最重要的不足并讨论了实现消除目标所需要的新工具和策略。从该次会议的演讲和讨论中形成的文稿以及额外邀请的文章，一共 27 篇组成了一期主题是“血吸虫病研究：提供用于消除的工具”的专辑。在这些文章中，探讨了几个与消除有关的关键问题，如血吸虫病是否能消除、是否需要非常高敏感性的诊断技术、消除阶段预防性化疗的作用、我们是否还需要吡喹酮之外的新药物等等。由于全球血吸虫病流行区的差异性，需要升级 WHO 的策略以纳入一些新的和差异性的指南。

Translated from English version into Chinese by Men-Bao Qian

CHINESE CENTER FOR DISEASE CONTROL AND PREVENTION
NATIONAL INSTITUTE OF PARASITIC DISEASES

Elimination de la schistosomiase: les outils nécessaires

Robert Bergquist, Xiao-Nong Zhou, David Rollinson, Jutta Reinhard-Rupp, Katharina Klohe

Résumé
Malgré les efforts menés à l'échelon mondial en vue de freiner le taux de schistosomiase, ce dernier reste de manière préoccupante élevée et le nombre total d'années de vie corrigée du facteur d'invalidité (AVCI) dû à cette infection a augmenté. Toutefois, ce taux est lié à la prise en compte de certains symptômes cliniques « subtiles » lors du calcul de l'AVCI, du moment où la morbidité grave n'existe plus dans de nombreux pays. Du coup, la stratégie actuelle est passée du fait de cibler la morbidité à l'élimination de l'infection en tant que problème de santé publique, afin de répondre à la feuille de route de l'Organisation Mondiale de la Santé (OMS) pour la lutte mondiale contre les maladies tropicales négligées (MTN) et la résolution WHA65.21. Ces mises au point ont

conduit à la première conférence du groupe de travail de recherche de l'Alliance Mondiale de la Schistosomiase (AMS) sur la recherche et le développement, organisée en Juin 2016 à Shanghai, République Populaire de Chine. L'avancée actuellement réalisée dans la lutte contre la schistosomiase a été examinée, tout en identifiant les lacunes operationnelles les plus pressantes en matière de recherche et discutant de nouveaux outils et stratégies nécessaires pour faire de l'élimination une chose concrète. Les articles émanant des exposés et discussions lors de cette réunion, de concert avec quelques manuscrits sollicités, ont été recueillis dans un numéro spécial constitué de 27 documents en tout, intitulé « Recherche sur la schistosomiase: Fournir les outils nécessaires pour l'Elimination ». Plusieurs questions cruciales liées à l'élimination de la maladie sont abordées dans ces communications, p.eg., la schistosomiase peut-elle être éliminée?, y-a-t-il besoin de diagnostic hautement sensible?, quel est le rôle de la chiomiothérapie préventive dans la phase d'élimination?, avons-nous besoin de nouveaux médicaments outre praziquantel?, etc. En raison de l'hétérogénéité des zones endémiques dans le monde, les mesures de l'OMS doivent être modérées, instituant ainsi de nouvelles et différenciées recommandations.

Translated from English version into French by Kokouvi Kassegne

Ликвидация шистосомоза: необходимые инструменты

Robert Bergquist, Xiao-Nong Zhou, David Rollinson, Jutta Reinhard-Rupp, Katharina Klohe

Реферат

Несмотря на глобальные усилия по сдерживанию распространения шистосомоза, он по-прежнему крайне высок, и общее число скорректированных на инвалидность лет жизни (DALYs) из-за того, что эта инфекция возросла. Однако, это связано с включением некоторых тонких клинических симптомов, в настоящее время учитываются при расчете результата DALY, в то время как тяжелая инвалидизация исчезла во многих странах. В результате, нынешняя стратегия изменилась от таргетирования заболеваемости для ликвидации инфекции в качестве проблемы общественного здравоохранения Всемирной организации здравоохранения (ВОЗ) "дорожной карты" для глобального управления забытых тропических болезней (NTDs) и резолюции WHA65.21. Эти события привели к первой конференции глобального Альянса Шистосомоз (GSA) исследовательской рабочей группы по исследованиям и развитию, состоявшейся в июне 2016 года в Шанхае, КНР. Нынешний прогресс в борьбе с шистосомозом состоится в том, чтобы выявлять наиболее актуальных потребностей в области операционных исследований и обсуждать новые инструменты и стратегии, чтобы сделать исключение стало реальностью. Статьи, исходящие из лекций и дискуссий, состоявшихся в ходе этой встречи, вместе с некоторыми дополнительными специальными документами, были собраны в специальном выпуске под названием исследования Шистосомоз. Предоставление необходимых средств для ликвидации, в том числе и 27 документов. Несколько важных вопросов, связанных с устранением заболевания рассматриваются в этих сообщениях, например, шистосомоз может быть ликвидирован?; Не требует высокочувствительной диагностики?; Какова роль превентивной химиотерапии в стадии ликвидации?; Нужны ли нам новые лекарства в дополнение к празиквантел (praziquantel)?, и т. д. Из-за неоднородности эндемичных районов в мире, политики в ВОЗ, возможно, намеренны обновить создание новых, дифференцированных принципов.

Translated from English version into Russian by Hao-Qi Zhang

Eliminación de la schistosomiasis: las herramientas necesarias

Robert Bergquist, Xiao-Nong Zhou, David Rollinson, Jutta Reinhard-Rupp, Katharina Klohe

Resumen

A pesar de los esfuerzos en todo el mundo para frenar la prevalencia de la schistosomiasis, ésta sigue siendo alarmantemente alta y el número global de años de vida ajustados por discapacidad (AVAD) debido a esta infección ha aumentado. Sin embargo, esto se debe a la inclusión de algunos síntomas clínicos "sutiles" que ahora se incluyen al calcular la puntuación AVAD, mientras que la morbilidad severa ha desaparecido en muchos países. Como resultado, la estrategia actual ha cambiado de dirigir la morbilidad a la eliminación de la infección como un problema de salud pública para cumplir con la hoja de ruta de la Organización Mundial de la Salud (OMS) para el control global de las enfermedades tropicales desatendidas (ETD) y la resolución WHA65.21. Estos acontecimientos llevaron a la primera conferencia del grupo de trabajo sobre investigación y desarrollo de la Alianza Mundial contra la Schistosomiasis (AMS) celebrada en junio de 2016 en Shanghai, República Popular de China. Se revisó el progreso actual en el control de la schistosomiasis identificando las

lagunas de investigación operacional más urgentes y discutiendo las nuevas herramientas y estrategias necesarias para hacer realidad la eliminación. Los artículos que emanan de las conferencias y discusiones durante esta reunión, junto con algunos artículos adicionales invitados, han sido recopilados en un fascículo especial titulado "Investigación sobre Schistosomiasis: Proporcionando las Herramientas Necesarias para la Eliminación", que consta de 27 artículos en total. En estas comunicaciones se abordan varias cuestiones críticas relacionadas con la eliminación de la enfermedad, por ejemplo, se puede eliminar la schistosomiasis?; Se requieren diagnósticos altamente sensibles?; Cuál es el papel de la quimioterapia preventiva en la fase de eliminación?; Necesitamos nuevos fármacos además del praziquantel?, etc. Debido a la heterogeneidad de las áreas endémicas en el mundo, es posible que sea necesario mejorar las políticas de la OMS estableciendo nuevas directrices diferenciadas.

Translated from English version into Spanish by Màrius V. Fuentes
